# Acupuncture for the Treatment of Dry Eye: A Multicenter Randomised Controlled Trial with Active Comparison Intervention (Artificial Teardrops)

**DOI:** 10.1371/journal.pone.0036638

**Published:** 2012-05-17

**Authors:** Tae-Hun Kim, Jung Won Kang, Kun Hyung Kim, Kyung-Won Kang, Mi-Suk Shin, So-Young Jung, Ae-Ran Kim, Hee-Jung Jung, Jin-Bong Choi, Kwon Eui Hong, Seung-Deok Lee, Sun-Mi Choi

**Affiliations:** 1 Acupuncture, Moxibustion & Meridian Research Centre, Korea Institute of Oriental Medicine, Daejeon, South Korea; 2 Department of Cardiovascular and Neurologic Diseases, College of Oriental Medicine, Graduate School, Kyung Hee University, Seoul, South Korea; 3 Department of Acupuncture and Moxibustion, College of Oriental Medicine, Kyung Hee University, Seoul, South Korea; 4 Division of Clinical Medicine, School of Korean Medicine, Pusan National University, Gyeongsangnam-do, South Korea; 5 Department of Oriental Rehabilitation Medicine, Dongshin University, Gwangju, South Korea; 6 Department of Acupuncture and Moxibustion, Daejeon University, Daejeon, South Korea; 7 Department of Acupuncture and Moxibustion, Dongguk University, Goyang, South Korea; Oregon Health & Science University, United States of America

## Abstract

**Purpose:**

To evaluate the effects of acupuncture compared to a control group using artificial tears.

**Methods:**

*Setting & design:* multicenter randomised controlled trial (three local research hospitals of South Korea). *Study Population:* 150 patients with moderate to severe dry eye. *Intervention:* Participants were randomly allocated into four weeks of acupuncture treatment (bilateral BL2, GB14, TE 23, Ex1, ST1, GB20, LI4, LI11 and single GV23) or to the artificial tears group (sodium carboxymethylcellulose). *Main Outcome Measure(s):* The ocular surface disease index (OSDI), tear film break-up time (TFBUT), Schirmer Ι test, visual analogue scale (VAS) for self-assessment of ocular discomfort, general assessment (by both acupuncture practitioners and participants) and quality of life (QOL) through the Measure Yourself Medical Outcome Profile-2 (MYMOP-2).

**Results:**

There was no statistically significant difference between two groups for the improvement of dry eye symptoms as measured by OSDI (MD −16.11, 95% CI [−20.91, −11.32] with acupuncture and −15.37, 95% CI [−19.57, −11.16] with artificial tears; P = 0.419), VAS (acupuncture: −23.84 [−29.59, −18.09]; artificial tears: −22.2 [−27.24, −17.16], P = 0.530) or quality of life (acupuncture: −1.32 [−1.65, −0.99]; artificial tears: −0.96 [−1.32, −0.6], P = 0.42) immediately after treatment. However, compared with artificial tears group, the OSDI (acupuncture: −16.15 [−21.38, −10.92]; artificial tears: −10.76 [−15.25, −6.27], P = 0.030) and VAS (acupuncture: −23.88 [−30.9, −16.86]; artificial tears: −14.71 [−20.86, −8.55], P = 0.018) were significantly improved in the acupuncture group at 8 weeks after the end of acupuncture treatment. TFBUT measurements increased significantly in the acupuncture group after treatment.

**Conclusions:**

Acupuncture may have benefits on the mid-term outcomes related to dry eye syndrome compared with artificial tears.

**Trial registration:**

ClinicalTrials.gov NCT01105221.

## Introduction

Dry eye syndrome is a common ophthalmologic disorder causing ocular discomfort in daily life. An increased knowledge of dry eye pathology has changed the definition of dry eye syndrome from describing a trivial ocular disorder related to secretion deficiency or excess tear evaporation to detailing a multi-factorial disease, which may involve chronic inflammation or tear film instability [1]. According to previously published literature, the overall prevalence of dry eye syndrome is estimated to be 5 to 35 percent in various populations [2]. Moreover, the incidence is now increasing, which may be related to changes in life style and working environments, increased average life expectancies and usage of medical interventions that can cause dry eye syndrome, including laser-assisted in situ keratomileusis (LASIK) surgery, radiation therapy, contact lenses and medications such as antihistamines or diuretics [1].

Recent studies have suggested that dry eye syndrome may pose a considerable economic burden on the patient and on society [3], [4]. Patients with dry eye syndrome not only have ocular discomfort but also visual disturbances; therefore, the impact is significant, affecting individual daily activities such as driving and reading as well as social functioning and productivity [4]. Many treatment options have been suggested, but artificial tears are the most widely chosen as lubricants or supplements for tear deficiency [5].

Acupuncture is one of the oldest interventions in East Asian countries. However, clinical evidence for the effectiveness of acupuncture has been provided for only a small number of diseases [6]. In the ophthalmologic field, recent studies have suggested that acupuncture may be helpful for several conditions including glaucoma [7] and amblyopia [8]. The efficacy and effectiveness of acupuncture for dry eye syndrome has also been explored in recent decades, but rigorous clinical trials have been needed to confirm the effectiveness of this intervention [9]. In this context, we tested the effectiveness of acupuncture compared to artificial tears as a control, an active treatment for dry eyes with high methodological rigor. A cost-effectiveness study and qualitative research were conducted simultaneously [10].

## Methods

The protocol for this trial and supporting CONSORT checklist are available as supporting information; see Checklist S1 and Protocol S1. This was a randomised, active controlled, parallel designed trial. The study protocol has been previously published [10]. This multicentre trial was conducted in three clinical research centres of South Korea from June to November 2010: Korea Institute of Oriental Medicine (Daejeon University Hospital), DongGuk University Ilsan Oriental Hospital and Dongshin University Gwangju Oriental Hospital. The research protocol was approved before study onset by the institutional review board (IRB) of each participating hospital. Participants were independently recruited by each centre through advertisements in local newspapers. The protocol was registered with ClinicalTrials.gov (Identifier: NCT01105221) before participant enrolment. Written informed consent was obtained from all of the participants.

Patients aged nineteen to sixty five years old with aggravating dry eye symptoms in a single eye or in both eyes were recruited [1]. Physicians and ophthalmologists assessed participant eligibility [11]. Inclusion criteria were based on the following ophthalmologic tests: a tear film break-up time (TFBUT) below 10 seconds and a Schirmer Ι test (with application of alcaine, a local anaesthetic) value below 10 mm/5 minutes [12]. These ophthalmologic tests were performed by the ophthalmologists who did not know the allocation results. Participants with several conditions were excluded: pathological changes of the eye, Stevens-Johnson syndrome, external injuries and eye-surgery history affecting dry eye. Participants who had been taking or needed active treatment for dry eyes were also excluded, as were patients with punctal occlusion history or current usage of anti-inflammatory eye drops. Contact lens use was prohibited throughout the participation period.

Participants were allocated into either acupuncture or artificial tears evenly. Random numbers were generated through computerised block-randomisation with the SAS package (SAS® Version 9.1, SAS Institute, Inc., Cary, NC) by separate statistician. Opaque assignment envelopes with consecutive numbers for each centre were used for allocation concealment. The necessary sample size was calculated from the results of previous studies regarding the effects of acupuncture [11], [13] and artificial tears [14]. The mean difference (standard deviation) of ocular surface disease index (OSDI) after acupuncture treatment was 17.61 (15.61), and after artificial tears (sodium carboxymethylcellulose), it was 11.3 (6.3) [13], [14]. Anticipating a 20% dropout rate, a total of 150 participants was recruited and was evenly assigned to each centre (50 participants in each centre).

### Interventions

#### Acupuncture Treatment Group

Acupuncture was administered according to the theory of traditional Korean medicine (TKM) without using lubricants. An expert committee composed of clinical experts and researchers working on acupuncture research or ophthalmologic practice of TKM decided on acupuncture points and needling methods based on published literature and textbooks about acupuncture for ophthalmologic diseases or dry eye syndrome [9], [15]. Certified practitioners with at least 7 years of TKM education and 3 years of clinical experience performed the acupuncture treatment. To reduce non-specific effects originating from the close relationship between patients and practitioners, interactions were strictly limited [16].

Seventeen acupuncture points (bilateral BL2, GB14, TE 23, Ex1, ST1, GB20, LI4, and LI11 and single GV23), located according to the WHO Standard Acupuncture Point Locations in the Western Pacific Region, were treated with 0.20×30-mm disposable acupuncture needles (Dongbang Co., Korea) [17]. The depths of inserted needles differed but were approximately 0.6 to 3 cm for the acupuncture points at the face and head (BL2∶1.5 to 3 cm; GB14∶0.9 to 1.5 cm; TE23∶1.5 to 3 cm; Ex1∶1.5 to 3 cm; ST1∶0.6 to 0.9 cm; GV 16∶0.9 to 1.5 cm) and 3 to 4.5 cm for points of hand (LI4) and arm (LI11). Each acupuncture needle was twisted until patient felt a ‘deqi’ sensation and retained for 20 minutes before removal. Participants had acupuncture treatments three times per week for four weeks (a total of 12 treatments).

#### Artificial Tears Group

Preservative-free single-use artificial teardrops (0.5% sodium carboxymethylcellulose) were provided, and participants were advised to use them as needed (at least once per day) for four weeks. A diary of both the frequency and quantity of drops used was collected at every visit. In both groups, other treatments for dry eyes were forbidden during the four weeks of treatment. However, during the follow-up period, participants were allowed to use any kind of treatment for dry eyes, and participant reports on treatment usage were requested at every visit.

#### Outcome Assessment

Outcome assessment included two aspects, subjective ophthalmologic tests and objective questionnaires for both ocular symptoms and quality of life related to dry eyes. The primary outcome was the difference in OSDI changes between the two groups. The secondary outcomes were the differences in 100 mm VAS for the ocular discomfort, quality of life questionnaire using the Measure Yourself Medical Outcome Profile-2 (MYMOP-2), TFBUT, Schirmer Ι test (with anaesthesia) score and adverse event rate of acupuncture treatment and artificial tears usage. Both eyes were assessed for the evaluation of TFBUT and Schirmer Ι test, respectively. Outcomes were assessed 13 weeks after the first visit.

OSDI is a validated questionnaire consisting of twelve questions for evaluating ocular symptoms and worsening conditions related to dry eyes [18]. Each question has a score between zero and four, where zero indicates “none of the time” and four indicates “all of the time”. The OSDI score was calculated according to the following formula: OSDI = [(sum of scores for all questions answered)*100]/[(total number of questions answered)*4]. The score ranges from 0 to 100, and higher scores represent a more severe dry eye state. The minimal clinically important difference (MCID) of OSDI for dry eye syndrome is suggested to be 7.3 to 13.4 points in severe dry-eye patients [19]. A version translated into the Korean language was used [20].

A 100 mm VAS for self-assessment of ocular discomfort was reported by participants. Ocular symptoms related to dry eye (e.g., ocular itching, foreign body sensation, burning, pain and dryness, blurred vision, sensation of photophobia, ocular redness, and sensations of tearing) were quantified and summarised in a standard 100 mm VAS scale.

The QOL section of the MYMOP-2 was adopted for assessing dry eye-related QOL [21], [22]. A seven-point Likert scale (from zero as ‘excellent’ to six as ‘worst’) was used for the assessment of QOL grade. The question was “During last week, how would you express your quality of life related to dry eyes, overall?”.

Tear film break-up time (TFBUT) is a test for assessing tear film stability [23], [24]. Sodium fluorescein (2.5%) was applied to both eyes, and the interval between the blink of eyes and the first appearance of a dry spot or disruption in the tear film was measured. If TFBUT is below 10 sec, it suggests at least a moderate severity of dry eyes [1], [12].

The Schirmer Ι test (with anaesthesia) is a diagnostic method to measure the basic quantity of tear secretion [12]. After application of local anaesthesia, Schirmer test paper (Color Bar, Eagle Vision, USA) was placed in the lateral third of the lower eyelids for 5 minutes with closed eyes. If the Schirmer test result is below 10 mm/5 min, it also suggests at least a moderate severity of dry eyes [1], [12].

General improvements of dry eye-related symptoms were assessed by practitioners and participants using a five-grade Likert scale: excellent, good, fair, poor and aggravation.

For the evaluation of safety issues, we assessed adverse events rates of acupuncture and artificial tears. If unexpected responses happened, the type and frequency were collected. The type and frequency of adverse events were reported for each group. According to the criteria of the WHO Toxicity Grading Scale for Determining The Severity of Adverse Events, the severity of the adverse event was evaluated by practitioners as grade 1 (mild) to grade 4 (life threatening) [25]. OSDI, VAS and quality of life were assessed by separate outcome assessors who did not perform acupuncture treatment. TFBUT and Schrmir Ι test were evaluated by ophthalmologists.

#### Statistics

To determine the differences between the acupuncture and artificial tears groups, the changes in values from baseline were compared at each visit on an intention-to-treat basis at a 95% significant level. Missing data of dropped-out participants were assigned by the last observation carried forward (LOCF) method. Participant expectations of acupuncture treatment for dry eye symptoms were collected with a nine-point Likert scale and were compared between the two groups [26]. Key baseline characteristics including risk factors for dry eyes such as computer use, age, occupational environment, contact lens usage, etc. were evaluated for the difference between two groups. ANCOVA (Analysis of Covariance) were used for continuous outcomes such as OSDI score, TFBUT, Schirmer test result, QOL and VAS for self-assessment of ocular discomfort, adjusted for baseline values and research centres as covariates. Adjusted differences, which were calculated from the ANCOVA model, were reported at each visit to estimate the effect size. Chi-squared tests were used for dichotomous outcomes such as general improvements of dry eye-related symptoms and differences in the usage of additional treatment during follow-up period. As suggested in the study protocol, repeated measures of analysis of variance for OSDI were performed to show trend changes. Statistical analyses were conducted using the SAS statistical package.

## Results

**Figure 1:**
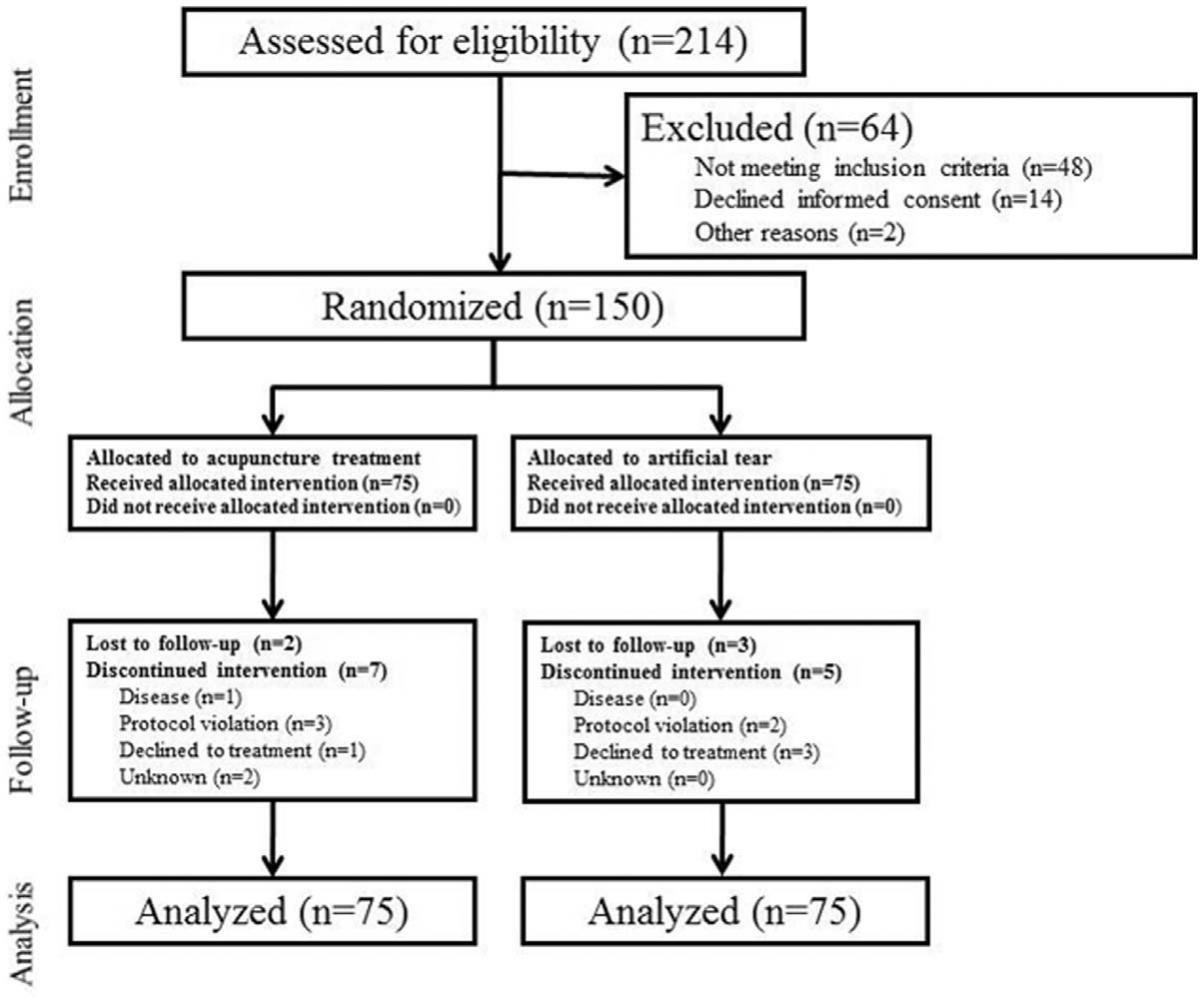
Study flow chart.

A total of 214 participants were assessed for eligibility at the three research centres. There were 150 dry-eye patients who met the inclusion criteria and were equally allocated into the acupuncture and artificial tears groups. Nine participants from the acupuncture group and 8 from the artificial tears group dropped out during the study (figure 1). In acupuncture treatment group, 2 participants were lost to follow up and 7 participants discontinued treatment because of acute keratitis (1), protocol violation (3), usage of prohibited treatments such as acupuncture in other clinic, application of steroid or anti-inflammatory drug (3), pain related to the acupuncture (1) and unknown reason (1). In the control group, 3 participants were lost to follow up and 5 discontinued participation because of protocol violation i.e. infringement of visiting schedule (2) and refusal of artificial tear usage due to inconvenience (3).

The baseline characteristics did not show important imbalances between the two groups, with similar risk factors for dry eyes such as time watching television and computer use, age, occupational environment, contact lens use and smoking [1]. Many participants had used artificial tears for controlling dry eye symptoms before study participation (table 1). Patient expectations for the effectiveness of acupuncture treatment for dry eyes did not differ between the two groups (Wilcoxon rank summed test, P = 0.8817).

During the 4-week treatment period, the control participants applied artificial tears an average of 2.79 times per day (95% CI [2.50, 3.08]) and used a total of 38.87 [36.24, 31.50] units of artificial teardrops.

**Table 1:** Baseline characteristics (intention-to-treat population).

	Acupuncture group	Artificial tears group
Characteristics	(n = 75)	(n = 75)
**Age (year, mean (SD))**	47.95 (11.11)	46.05 (13.10)
**Sex M/F (frequency)**	22/53	19/56
**Menopause (frequency)**	26	25
**Symptom duration (frequency, mean (SD))**	6.00 (5.69)	5.91 (4.97)
**Computer or TV use (hour/week, mean (SD))**	16.87 (15.53)	18.61 (16.80)
**Contact lens wearer (frequency)**	7	6
**Smoker (frequency)**	7	7
**Full time-outdoor workers/full time-indoor workers (frequency)**	36/4	27/3
**>10 years of school education (frequency)**	65	67
**Previous treatment (frequency)**		
** Artificial tears use**	33	44
** Other treatment use**	5	3
** No-treatment**	37	28
**Past history related to dry eye (frequency)**		
** Sjogren syndrome**	0	0
** Keratitis**	7	10
** Cataract**	2	3
** Glaucoma**	2	1
** LASIK surgery**	1	6
** Blepharitis**	2	3

### OSDI

**Figure 2:**
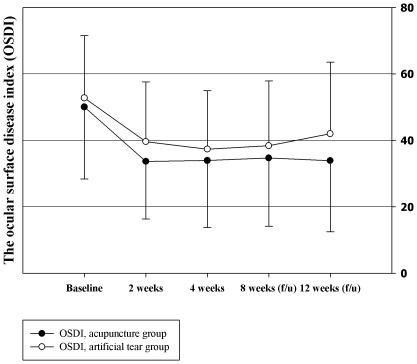
Ocular surface disease index (OSDI).

Changes in OSDI from baseline values did not show significant differences between the two groups after 2 weeks of treatment, with the acupuncture group showing a change of -16.38 (95% CI [−20.7, −12.06]) and the artificial tears group showing a change of −13.1 (95% CI –[17.68, −8.52]), as tested by ANCOVA, P = 0.058. After 4 weeks of treatment, the change in OSDI was −16.11, [−20.91, −11.32] and −15.37 [−19.57, −11.16] for the acupuncture and artificial tears groups, respectively (P = 0.419). At 4 weeks after the end of acupuncture, the change in OSDI was −15.35 [−19.82, −10.88] and −14.38 [−18.84, −9.92] for the acupuncture and artificial tears groups, respectively (P = 0.416). However, significant improvement in OSDI in the acupuncture group was reported at the 8-week after acupuncture treatment (acupuncture: −16.15 [−21.38, −10.92]; artificial tears: −10.76 [−15.25, −6.27], P = 0.030). As the MCID for severe dry eyes is estimated to be 7.3 to 13.4 [19], these results can be interpreted to show that both acupuncture and artificial tears improved the symptoms of dry eyes during the treatment period, but the therapeutic effect was maintained longer in the acupuncture group (figure 2). The adjusted difference of OSDI was −0.75 [−4.22, 2.72] after 4 weeks of acupuncture treatment, and it increased in favour of acupuncture to −0.98 [−4.08, 2.14] at 4 weeks and to −5.39 [−8.62, −2.16] at 8 weeks after the end of treatment (table 2). From the result of repeated measure analysis of variance, no significant differences in the trends of changes in OSDI value were observed between the two groups (p = 0.31).

**Table 2:** Primary and secondary outcomes at each visit.

	Acupuncture group (n = 75)					Artificial tears group (n = 75)					Adjusted difference†			
	Mean (SD)					Mean (SD)					(95% CI)			
	Baseline	During treatment		Follow up		Baseline	During treatment		Follow up		During treatment		Follow up	
		2 weeks	4 weeks	8 weeks	12 weeks		2 weeks	4 weeks	8 weeks	12 weeks	2 weeks	4 weeks	8 weeks	12 weeks
**OSDI**	50.05 (21.63)	33.67 (17.35)	33.94 (20.16)	34.70 (20.57)	33.90 (21.42)	52.75 (18.79)	39.65 (17.94)	37.38 (17.61)	38.37 (19.51)	41.99 (21.54)	−3.28(−7.06, 0.50)	−0.75(−4.22, 2.72)	−0.98(−4.08, 2.14)	−5.39*(−8.62, −2.16)
**VAS**	66.67 (19.18)	48.77 (21.44)	42.83 (23.74)	47.29 (22.90)	42.79 (26.88)	67.52 (17.31)	46.23 (17.92)	45.32 (20.24)	51.07 (23.07)	52.81 (24.12)	3.40(−0.52, 7.32)	−1.64(−5.17, 1.89)	−2.92(−7.99, 2.15)	−9.17*(−13.70, −4.65)
**QOL**	4.04 (1.20)	3.01 (1.34)	2.72 (1.22)	2.96 (1.31)	2.95 (1.47)	4.08 (1.21)	3.12 (1.11)	2.88 (1.15)	3.12 (1.30)	3.32 (1.37)	−0.07(−0.31, 0.18)	−0.12(−0.40, 0.15)	−0.12(−0.37, 0.13)	−0.34(−0.58, −0.09)
**BUT**	6.19 (2.18)		6.80 (2.25)		6.68 (2.85)	6.01 (1.98)		5.89 (2.02)		5.89 (1.99)		0.73*(0.32, 1.14)		0.61(0.14, 1.07)
**Schirmer**	4.49 (2.56)		4.88 (3.70)		5.95 (5.02)	4.16 (2.66)		4.95 (4.34)		5.28 (4.07)		−0.40(−0.75, −0.05)		0.33(0.02, 0.64)

ANCOVA was used for the statistical analysis of changes from baseline in each outcome between two groups. *P<0.05.

† All the outcomes were adjusted for baseline values and clinical research centres. Negative values of adjusted difference in OSDI, VAS and QOL (positive difference in BUT and Schirmer) are in favour of acupuncture group.

### VAS

**Figure 3:**
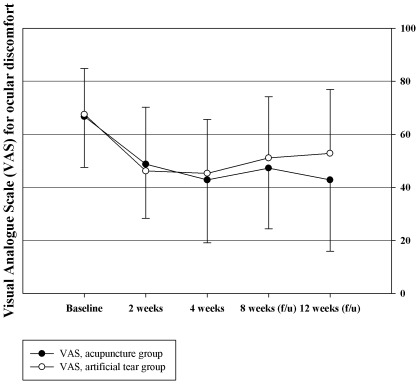
Visual analogue scale (VAS) for self-assessment of ocular discomfort.

Changes in VAS showed a pattern similar to that of OSDI. Statistically significant improvements did not appear at 2 weeks (−17.89 [−23.27, −12.51] in the acupuncture group and −21.29 [−26.19, −16.4] in the artificial tears group, P = 0.359) or 4 weeks of treatment (acupuncture: −23.84 [−29.59, −18.09]; artificial tears −22.2 [−27.24, −17.16], P = 0.530) or at 4 weeks after treatment (acupuncture: −19.37 [−25.51, −13.24]; artificial tears: −14.71 [−20.86, −8.55], P = 0.327). However, significant changes were reported in the acupuncture group at 8 weeks after acupuncture treatment compared with control group (acupuncture: −23.88 [−30.9, −16.86]; artificial tears: −14.71 [−20.86, −8.55], P = 0.018). The adjusted difference of VAS was −1.64 [−5.17, 1.89] for the acupuncture group after 4 weeks of treatment, and it increased to −2.92 [−7.99, 2.15] at 4 weeks and to −9.17 at 8 weeks after treatment (table 2, figure 3).

### QOL

Changes in QOL from baseline values did not show significant differences between the two groups after 2 weeks of treatment (−1.03 [−1.34, −0.71] in the acupuncture group and −1.2 [−1.53, −0.87] in the artificial tears group, as tested by ANCOVA, P = 0.63), after 4 weeks of treatment (acupuncture: −1.32 [−1.65, −0.99]; artificial tears: −0.96 [−1.32, −0.6], P = 0.42), at 4 weeks after treatment (acupuncture: −1.09 [−1.47, −0.72]; artificial tears: −0.96 [−1.32, −0.6], P = 0.47) or at 8 weeks after treatment (acupuncture: −1.09 [−1.47, −0.72]; artificial tears: −0.76 [−1.1, −0.42], P = 0.11, table 2).

### TFBUT

**Figure 4:**
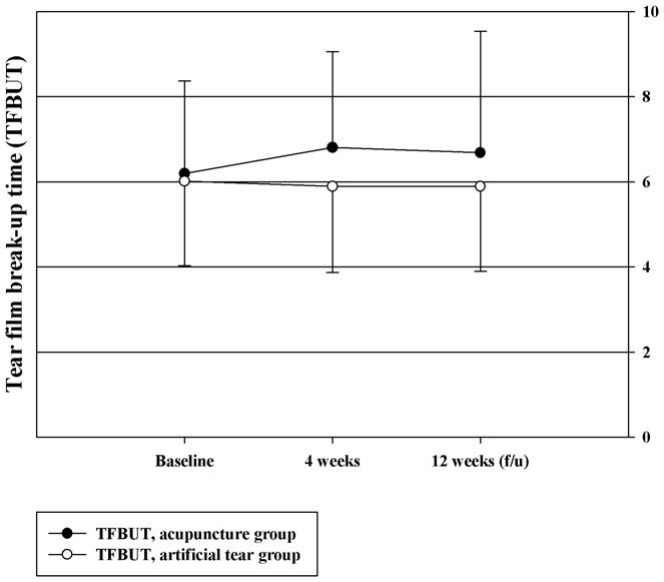
Tear film break-up time (TFBUT).

TFBUT increased significantly in the acupuncture group after 4 weeks of treatment compared to the artificial tears group (figure 4). The change from the baseline of TFBUT in the left eye was 0.46 [−0.04, 0.96] in the acupuncture group and −0.37 [−0.95, 0.21] in the artificial tears group (P = 0.005), and that in the right eye was 0.59 [0.05, 1.14] and −0.09 [−0.63, 0.44] (P = 0.017), respectively. At 8 weeks after acupuncture, TFBUT in the right eye showed a significant increase in the acupuncture group (acupuncture: 0.72, [0, 1.43]; artificial tears: −0.23 [−0.76, 0.31], P = 0.017) but TFBUT in the left eye did not (acupuncture: 0.3 [−0.38, 0.97]; artificial tears: −0.21 [−0.81, 0.39], P = 0.151). Adjusted differences between the two groups (calculated using representative values from the eye with the higher baseline value) were 0.73 [0.32, 1.14] after 4 weeks (P = 0.007, tested by ANCOVA) and 0.61 [0.14, 1.07] at 8 weeks after acupuncture (P = 0.054).

### Schirmer Ι Test Scores

**Figure 5:**
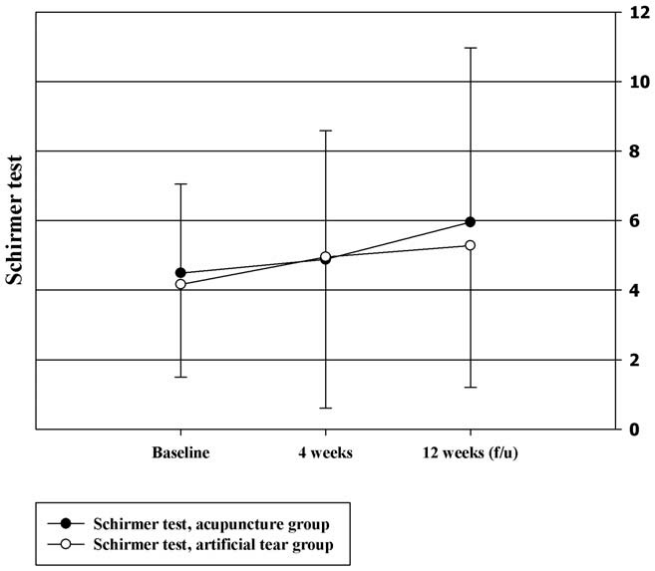
Schirmer Ι test.

There were no statistically significant differences in the Schirmer Ι test score changes of both eyes between the two groups after 4 weeks of treatment (left eye: 0.59 [−0.22, 1.4] in the acupuncture group and 0.55 [−0.64, 1.73] in the artificial tears group, tested by ANCOVA, P = 0.997; right eye, acupuncture: 0.59 [0.05, 1.14] and artificial tears: −0.09 [−0.63, 0.44], P = 0.904) or after 8 weeks after treatment (left eye, acupuncture: 1.77 [0.4, 3.13] and artificial tears: 0.61 [−0.54, 1.76], P = 0.192; right eye, acupuncture: 1.45 [0.13, 2.77] and artificial tears: 1.4 [0.11, 2.69], P = 0.857, figure 5).

### General Improvements of Dry Eye-related Symptoms

More participants in the acupuncture group experienced general improvements than did those in the artificial tears group; however, there were no significant differences between the two groups (P = 0.060). In contrast, a statistically significant difference was reported between the two groups in the general improvements assessed by physicians (P = 0.001, table 3).

**Table 3:** General improvements of dry eye-related symptoms.

	Assessed by participants (n = 137)*					Assessed by physicians (n = 136)^†^				
Grade	E	G	F	P	A	E	G	F	P	A
Acupuncture	2(2.99%)	27(40.30%)	28(41.79%)	10(14.93%)	0(0%)	4(5.97%)	28(41.79%)	25(37.31%)	10(14.93%)	0(0%)
Artificial tears	2(2.86%)	17(24.29%)	26(37.14%)	24(34.29%)	1(1.43%)	0(0%)	17(24.64%)	22(31.88%)	27(39.13%)	3(4.35%)

Tested by Chi-squared test; *P = 0.060, ^†^P = 0.001; E: excellent, G: good, F: fair, P: poor, A: aggravation.

### Use of Additional Treatment During the Follow-up Period

During the follow-up period, 9 participants in the artificial tears group treated dry eyes continuously; 3 participants visited ophthalmic clinics and used prescribed lubricants or eye drops, and 6 participants applied over-the-counter artificial teardrops. In addition, 4 participants in the acupuncture group used artificial tears after acupuncture treatment; 1 participant visited an ophthalmic clinic, and the other 3 applied over-the-counter artificial tears.

### Adverse Events

Three cases of hematoma were reported in the acupuncture treatment group; 1 patient had moderate severity, and the others had mild severity. Among them, one participant declined further acupuncture treatment because of the hematoma and pain. In the other two cases, the hematomas completely disappeared in several weeks. Adverse events related to artificial tear usage was not reported.

## Discussion

This clinical trial assessed the effectiveness of a 4-week standard acupuncture treatment on the subjective and objective outcomes of dry eye syndrome. Although overall improvement was observed in both groups, there was no statistically significant difference in favour of acupuncture treatment for OSDI, VAS or quality of life during the treatment period or immediately after treatment. Improved OSDI and VAS scores, however, were sustained for 8 weeks in the acupuncture group, but not in the artificial tears group. Among the ophthalmologic tests, TFBUT increased significantly in the acupuncture group, but the Schirmer test results did not.

### Strength and Weakness

Previous research on dry eye syndrome has provided limited evidence of the effectiveness of acupuncture due to poor methodological quality and insufficient statistical power [9]. We previously conducted a case study to test the validity and availability of acupuncture [27] and an RCT using sham acupuncture as a control to evaluate the efficacy of acupuncture for dry eye syndrome [13]. From the results of these studies, we designed a clinical trial adopting an active control for assessing the effectiveness of acupuncture with a sufficient number of subjects for statistical analyses [10]. To increase the universality of the study results (i.e., external validity), three research hospitals located in different areas conducted this trial simultaneously. To the best of our knowledge, this is the first study that had the statistical power (calculated from previous studies) and methodological rigor to allow strong conclusions regarding the effectiveness of acupuncture for the treatment of dry eye syndrome.

Along with subjective outcomes, it has recently been suggested that objective outcomes should also be tested for the interventions that involve large non-specific effects such as acupuncture [28]. Therefore, we assessed both subjective and objective outcome measures simultaneously. To reduce detection bias, separate outcome assessors evaluated treatment effects, and practitioners were instructed to not have active interaction with their patients in the process of acupuncture treatment. However, despite these attempts, it is possible that the non-specific effects of acupuncture could not be completely avoided.

This study has several weaknesses. First, because the main outcomes showed the effectiveness of the acupuncture group 8 weeks after treatment, the follow-up period should have been longer to evaluate the sustained effects of acupuncture treatment. Considering the average symptom duration of dry eye syndrome, evaluating the long term effects would be necessary in future trials. Second, this study result may not completely reflect actual clinical practice situations. There are multiple variations of acupuncture treatment including the style of acupuncture, needling methods, point selection and treatment sessions [9]. We adopted a standardised acupuncture modality based on TKM theory, which differs from previous researchers in the style of acupuncture and treatment regimen [29]–[31]. We believe that different acupuncture methods result in different effects. To optimise acupuncture effects, the styles, needling details and other components involved in this intervention should be compared in future research. Third, the use of a control intervention might be considered a controversial issue. Our previous study comparing actual and sham acupunctures [13] did not prove the specific effects of acupuncture, which is the general anticipated outcome of acupuncture trials [32]. Literature review has suggested that sham acupuncture interventions might have larger effects than placebo drugs, and they are usually associated with a comparatively large non-specific effect, which is one of the major obstacles in evaluating the efficacy of acupuncture [33]. In this sense, comparative effectiveness trials have emerged as alternatives for evaluating the clinical value of acupuncture treatment in the real world [34], [35]. As an active treatment for dry eyes, artificial tears are generally recommended as a first-line therapy [36]. According to a survey report of ophthalmologists, preservative-free artificial tears were most frequently used for moderate to severe dry-eye patients among various treatment modalities in Korea [37]. From this perspective, comparing acupuncture and artificial tears is a basic step for establishing the effectiveness of acupuncture treatment for dry eyes, as we originally intended in this trial. Additionally, if there may be any confirmed benefit in favour of acupuncture and no evidence of harm, we suggest that acupuncture can be given to patients who want acupuncture or who did not show a good response to conventional treatment on the basis of these results [38]. Fourth, the frequency of the average usage of artificial tear was below four times a day which is suggested to be the standard dose of sodium carboxymethylcellulose, so participants in the control group used artificial tear relatively infrequently. However, participants were allowed to apply artificial tear freely for easing their dry eye symptoms without frequency limit. As a result, there was one participant who used tears 15 times a day at most. Suggesting that artificial tear is generally accepted for alleviating bothering symptoms related to dry eye, allowing different frequency of usage in accordance with dry eye severity might not contribute the effectiveness of artificial tear drop in this trial. Finally, although we adopted the results of objective ophthalmologic tests as screening criteria for moderate to severe dry eye syndrome, there is a possibility that false-positive or false-negative dry eye patients might have been included in this study. Recent studies about TFBUT and Schirmer test suggested that very limited utility of these tests might introduce bias for identifying patients with mild to moderate dry eye [39], [40]. However, considering that tear osmolarity, which is accepted as a good marker for dry eye, was not available because of the required laboratory instruments and the participants’ discomforts over collecting large amount of tear specimens, TFBUT and Schirmer test were the second best policy for this study [39].

### Meaning of the Study

This study indicates that acupuncture is not more effective than artificial tears for the treatment of dry eyes in the improvement of subjective ocular symptoms such as OSDI during the treatment period. However, this does not mean that acupuncture is not a good intervention for dry eye syndrome. Acupuncture may have benefits on the mid-term outcomes related to dry eye syndrome. Although statistically significant differences were not observed in the assessment of dry eye symptoms after 4 weeks of treatment, both treatments showed improvements in symptoms greater than the MCID for OSDI [19]. In addition, the improvement of OSDI was sustained until 8 weeks after treatment in the acupuncture group, but not in the artificial tears group. Furthermore, fewer patients visited ophthalmic clinics to manage dry eye symptoms in the acupuncture treatment group during the follow-up period. Apart from these subjective outcomes, TFBUT (which objectively reflects the stability of pre-corneal tear film) showed significant improvement in the acupuncture group. A comprehensive discussion about the effectiveness of acupuncture (including the results of the cost-effectiveness analysis and qualitative study) will be necessary to conclusively judge the benefits of this intervention.

### Unanswered Questions and Suggestions for the Future Research

Contrary to the temporal effect of artificial tear which works as a lubricant, acupuncture may act in other ways to improve dry eye syndrome. Acupuncture may reduce chronic inflammation of the ocular surface or accessory organs through the cholinergic anti-inflammatory pathway by enhancing vagus nerve activity [41]. Dry eye patients treated with acupuncture showed prolonged improvement in this trial and it might be explained by the effect of acupuncture of relieving pathologic cause of dry eye. In addition, ocular irritation, one of the disturbing symptoms of dry eye, can be decreased through the analgesic effects of acupuncture as well [42].

Previous research on dry eye syndrome has shown the benefits of acupuncture on the improvement of both objective (TFBUT, Schirmer and cornea fluorescent staining) and subjective (response rate) outcomes [29]–[31]. However, the results of this study indicate that there was no significant difference between the acupuncture and artificial tears groups after 4 weeks of treatment, and only TFBUT measurements improved significantly. This might be partially due to the heterogeneity in treatment durations and acupuncture treatment methods (including acupuncture points) utilised in the previous studies. However, the effect of acupuncture might be overestimated in these studies because of methodological flaws and inappropriate statistical power [43]. Generally, less rigorous trials are prone to exaggerate treatment effects [44] and studies with small sample sizes may underestimate it [45].

As with other medical interventions, acupuncture-related adverse events have been reported, but acupuncture is typically regarded as safe when applied by well-trained practitioners [46]. Recent observational studies have suggested that the frequency of adverse events related to acupuncture in trials and clinical practices ranges from 3.2% to 7.4% according to the population and treatment modalities [47]–[49]. Although serious adverse events did not occur in this study, 3 participants (4%) in the acupuncture group experienced adverse events related to their treatment in agreement with the general risk rate accompanying usual acupuncture practice. Because adverse events affect patient compliance to acupuncture, patients should be informed before treatment, and practitioners should adhere to safety guidelines on acupuncture use to avoid preventable errors.

## Supporting Information

Checklist S1 **CONSORT Checklist.**
(PDF)

Protocol S1 **Trial Protocol.** (published at open assess journal, Trials 2010)
(DOC)
